# ANCA-associated vasculitis in idiopathic pulmonary fibrosis: A case report and brief review of the literature

**DOI:** 10.1097/MD.0000000000029008

**Published:** 2022-03-04

**Authors:** Daniel Traila, Monica Steluta Marc, Camelia Pescaru, Diana Manolescu, Ovidiu Fira-Mladinescu

**Affiliations:** a XIIIth Department of Pulmonology, Center for Research and Innovation in Precision Medicine of Respiratory Diseases, “Victor Babes” University of Medicine and Pharmacy Timisoara, Timişoara, Romania; b Expert Centre for Lung Rare Diseases, Clinical Hospital of Infectious Diseases and Pneumophthisiology “Dr. Victor Babes” Timisoara, Timisoara, Romania.

**Keywords:** ANCA-associated vasculitis, idiopathic pulmonary fibrosis, microscopic polyangiitis

## Abstract

**Rationale:**

Idiopathic pulmonary fibrosis (IPF) is a progressive disease with poor prognosis. Patients with IPF represent a heterogeneous population with several described clinical phenotypes. More recently, the development of antineutrophil cytoplasmic antibody (ANCA)-associated vasculitis in IPF patients, with an incidence higher than that in the general population, has drawn attention.

**Patient concerns:**

A 64-year-old woman previously diagnosed with IPF presented to the emergency department with hemoptysis and hypoxemic respiratory failure.

**Diagnoses:**

High-resolution chest computed tomography revealed bilateral ground-glass opacities associated with areas of consolidation superimposed on the patient's fibrotic background pattern. Diffuse alveolar hemorrhage was confirmed by the presence of hemorrhagic bronchoalveolar lavage fluid. Hematological and biochemical investigations revealed an inflammatory syndrome, moderate anemia, and rapidly progressive glomerulonephritis. Serological analysis revealed perinuclear antineutrophil cytoplasmic antibody positivity and high levels of antimyeloperoxidase antibodies antibodies. The patient underwent kidney biopsy, which revealed necrotizing glomerulonephritis. Clinical and laboratory findings were diagnostic of microscopic polyangiitis with lung and renal involvement.

**Interventions:**

Cyclophosphamide in combination with methylprednisolone was administered as remission induction therapy. The maintenance therapy consisted of mycophenolate mofetil and prednisone.

**Outcomes:**

The patient achieved clinical, radiological, and serological remission within six weeks of treatment.

**Lessons:**

The association between IPF and ANCA-associated vasculitis may represent a distinct clinical phenotype. Autoimmune testing for ANCAs should be considered part of the diagnostic work-up and follow-up of patients with IPF because of the clinical and therapeutic implications of developing vasculitis in an already vulnerable patient.

## 1 Introduction

Idiopathic pulmonary fibrosis (IPF) is a progressive fibrosing interstitial pneumonia of unknown cause that occurs primarily in older adults, is limited to the lungs, and is characterized by the histopathological pattern of usual interstitial pneumonia (UIP). IPF is associated with poor prognosis, with a median life expectancy of 2.5 to 3.5 years after diagnosis.^[1]^ The diagnosis of IPF requires the exclusion of other known causes of interstitial lung disease, such as domestic and occupational environmental exposures, connective tissue disease, or drug toxicity.^[2]^ Familial IPF accounts for approximately 4% of all cases.^[3]^

Disease progression in an individual patient is difficult to predict, as there is a well-recognized clinical heterogeneity of IPF. Currently described clinical phenotypes with differences in clinical behavior include rapidly progressive IPF, familial IPF, combined pulmonary fibrosis and emphysema, pulmonary hypertension associated with IPF, and acute exacerbation of IPF.^[4,5]^

Few publications have reported the development of antineutrophil cytoplasmic antibody (ANCA)-associated vasculitis in patients with an established IPF diagnosis. ANCA-associated vasculitis is an immune-mediated group of diseases characterized by necrotizing vasculitis, which primarily affects the small blood vessels of the airway and kidneys. The major clinicopathological variants are granulomatosis with polyangiitis ( formerly known as Wegener granulomatosis), eosinophilic granulomatosis with polyangiitis ( formerly known as Churg-Strauss syndrome), and microscopic polyangiitis (MPA). ANCA-associated vasculitis reported in association with IPF is mostly represented by MPA with antimyeloperoxidase antibodies (anti-MPO).^[6]^

In this case report, we present a patient with IPF with subsequent development of systemic vasculitis and review the relevant literature regarding pulmonary fibrosis with ANCA-associated vasculitis. Awareness of this possible association is important, as the development of vasculitis in an already vulnerable IPF patient could represent an event with significant mortality.

## 2 Case presentation

**Figure 1:**
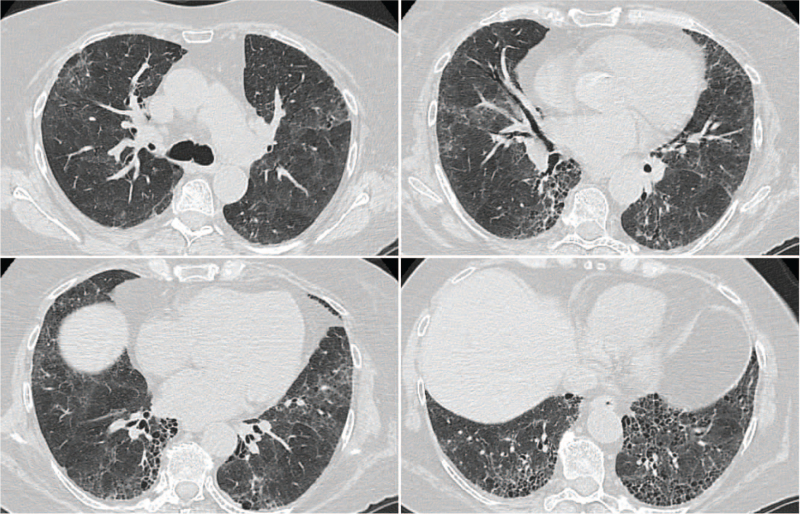
Chest HRCT: subpleural with basal predominance honeycombing, traction bronchiectasis, and mild ground-glass opacification superimposed on a fine reticular pattern. HRCT= high-resolution computed tomography.

A 64-year-old woman presented with progressive breathlessness on exertion and bibasilar inspiratory Velcro crackles on chest auscultation. Chest high-resolution computed tomography (HRCT) revealed changes consistent with the UIP pattern (Fig. 1). Pulmonary function testing demonstrated a restrictive ventilatory defect (FVC = 77% predicted) in association with a moderately reduced diffusing capacity of the lung for carbon monoxide (DLCO = 54% predicted).

The patient was a nonsmoker with no relevant environmental antigenic exposure or drug toxicity. Serological studies for collagen vascular disease (including rheumatoid factor, anticyclic citrullinated peptide, and antinuclear antibody titer and pattern) were negative. Bronchoalveolar lavage revealed a normal cytology.

Based on clinical background and radiological data, a diagnosis of IPF was established (UIP pattern without evidence of a known cause). The patient was started on antifibrotic therapy with nintedanib. Her IPF remained radiologically stable; however, there was a gradual decline in diffusing capacity of the lung for carbon monoxide to 50% predicted at 6 months.

**Figure 2:**
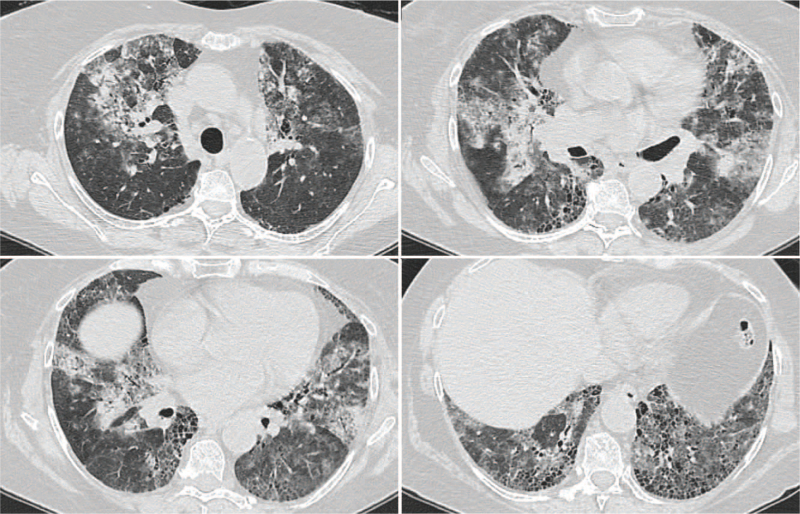
Chest HRCT: bilateral patchy ground-glass opacities and lobular areas of consolidation superimposed on the usual interstitial pneumonia background. HRCT= high-resolution computed tomography.

One year after IPF diagnosis, the patient presented to the emergency department with hemoptysis and hypoxemic respiratory failure. On admission, the patient was febrile (38.5°C) and exhibited tachypnea (26 bpm). She was hemodynamically stable with an oxygen saturation of 84% on room air, which increased to 96% with 100% oxygen. Evaluation by chest HRCT revealed new bilateral diffused ground-glass opacities associated with areas of consolidation superimposed on the already known patient's fibrotic background pattern (Fig. 2).

Considering the COVID-19 pandemic, the patient was evaluated for SARS-CoV-2. Both RT-PCR amplification of SARS-Cov-2 virus nucleic acid from a nasopharyngeal swab and COVID-19 serology (IgM and IgG detection by ELISA antibody test) results were negative.

Routine hematology and biochemistry investigations revealed inflammatory syndrome (C-reactive protein = 91.5 mg/L) and moderate normochromic, normocytic anemia (Hb = 9.9 g/dL). Rapidly progressive glomerulonephritis was identified by active urinary sediment on urinalysis, microscopic hematuria, and proteinuria (2746 mg/24 h) in the setting of rising blood urea nitrogen (100.7 mg/dL) and serum creatinine (2.51 mg/dL). Due to the presence of hemoptysis, diffuse alveolar opacities, and low Hb levels, diffuse alveolar hemorrhage was considered and confirmed by hemorrhagic bronchoalveolar lavage fluid.

The presence of both diffuse alveolar hemorrhage and rapidly progressive glomerulonephritis suggested pulmonary renal syndrome with a primary differential diagnosis including ANCA-associated vasculitis , Goodpasture syndrome, and systemic lupus erythematosus. Serological analysis confirmed perinuclear antineutrophil cytoplasmic antibody positivity and high levels of anti-MPO antibodies (77.2 U/mL). A kidney biopsy revealed necrotizing glomerulonephritis. Clinical and laboratory findings were suggestive of microscopic polyangiitis.

According to the European Vasculitis Study Group classification,^[7]^ the disease severity was graded as generalized active. The patient was administered cyclophosphamide in combination with high-dose glucocorticoids for induction and maintenance therapy. The full therapy consisted of 6 intravenous pulsed cyclophosphamide (15 mg/kg every 2 weeks for the first 3 pulses, followed by 3 pulses every 3 weeks) and 1 g of intravenous methylprednisolone for 3 consecutive days followed by 0.5 mg/kg/d oral prednisone with subsequent slow tapering to 0.25 mg/kg/d at 12 weeks. Antifibrotic treatment with nintedanib was continued. No treatment-related adverse events were observed.

The patient achieved clinical and serological remission within 6 weeks of diagnosis with renal function recovery, resolution of ground-glass opacities on chest HRCT, and normalization of anti-MPO antibody levels. Maintenance therapy consisted of mycophenolate mofetil (2 g/d) and prednisone (0.25 mg/kg/d). Mycophenolate mofetil was preferred in our patient with IPF, patient considering its potential benefit on lung function through its antiproliferative and antifibrotic effects.^[8]^ The patient remained in remission 6 months after the diagnosis of vasculitis.

## 3 Discussion

Evidence indicates that the prevalence of ANCA positivity in patients with IPF is higher than that in the general population. At the time of IPF diagnosis or during follow-up, up to 20% of patients present with ANCA positivity, without clinical manifestations of systemic vasculitis.^[9–12]^ The most frequently detected ANCAs are anti-MPO and much less often antiproteinase antibodies , antielastase, antilactoferrin, or nonspecific ANCA positivity. There are no differences in clinical background, laboratory results, or pulmonary function tests between the ANCA-negative and ANCA-positive IPF patients. One study of IPF patients from North American cohorts found that MPO positivity predominated in women, whereas all PR3-positive individuals were male.^[9]^ Studies from Japanese populations have found equal sex distributions.^[10,11,13]^

Chest HRCT studies in ANCA-positive IPF subjects showed a significantly higher prevalence of ground-glass opacities superimposed on the UIP background pattern (increased attenuation around honeycombing).^[9,13]^ These radiological findings translate into predominant lung inflammation, as lung biopsies of patients with ANCA positivity revealed more frequent interstitial inflammation, plasma cell infiltration, lymphoid follicles with germinal centers, and cellular bronchiolitis. ANCA-positive IPF patients may present more neutrophils in bronchoalveolar lavage fluid than patients without ANCA.^[13]^

In our patient, ground-glass opacity admixed with reticular abnormality and traction bronchiectasis were present on chest HRCT before the development of clinical vasculitis. This finding was considered part of the fibrotic process. The limitation of the present clinical case is the lack of ANCA evaluation at the baseline diagnosis of IPF and thus the impossibility of correlating the baseline radiological anomalies with a possible ANCA positivity.

A significant proportion of ANCA-positive IPF patients develop vasculitis. Up to 26% of anti-MPA-positive patients develop MPA with anti-MPO antibodies.^[9–12]^ Significantly few studies have reported the development of granulomatosis with polyangiitis in antiproteinase antibodies positive IPF patients.^[14]^ The most frequently reported clinical manifestations of MPA in the IPF population are rapidly progressive glomerulonephritis, mononeuritis multiplex, diffuse alveolar hemorrhage, and gastrointestinal involvement.^[9,12,15]^

Several studies have reported a lower incidence of MPA in patients with ANCA-positive IPF patients treated with corticosteroids.^[10,12]^ However, chronic use of corticosteroids is no longer recommended in IPF, as randomized trials have shown an increased risk of hospitalization and death associated with immunosuppressant therapy.^[3]^ There was no significant difference in survival between ANCA-positive and ANCA-negative IPF patients or between ANCA-positive patients treated or not treated with corticosteroids.^[9,12]^ Thus, the available data do not support the use of corticotherapy to prevent MPA development in ANCA-positive IPF patients, as benefits outweigh the risks.

The treatment of ANCA-associated vasculitis in patients with IPF follows international guidelines for vasculitis. Combination therapy with corticosteroids and cyclophosphamide is the mainstay of remission induction treatment for severe MPA. One study that compared the prognosis of the UIP pattern in MPO-ANCA nephritis patients with the prognosis of IPF patients found no significant difference in the median survival time.^[16]^

The pathophysiological mechanisms underlying the association between ANCA vasculitis and pulmonary fibrosis remain poorly understood. It remains unclear whether this association represents 2 distinct diseases with potential pathophysiological links or manifestations of the same disorder. IPF inducing ANCA-vasculitis is suggested by the higher age at presentation, similar to IPF alone, and already established pulmonary fibrosis preceding full vasculitis syndrome in the majority of patients. UIP is the most frequent radiological and histological pattern in ANCA-positive pulmonary fibrosis, and appears to be the same as that in IPF. Conversely, ANCA vasculitis can lead to pulmonary fibrosis through recurrent occult alveolar hemorrhage or direct fibrogenicity of anti-MPO antibodies.

## 4 Conclusion

IPF with ANCA positivity could represent a distinct phenotype characterized by ANCA-positive conversion (mostly anti-MPO), the presence of ground-glass attenuation around honeycombing on chest HRCT, and possible subsequent development of clinical vasculitis in an IPF patient. Autoimmune testing for ANCAs should be considered part of the diagnostic work-up and follow-up of IPF because of the clinical and therapeutic implications of developing vasculitis.

## Acknowledgment

The authors thank the patient for granting permission to publish this manuscript.

## Author contributions

**Conceptualization:** Ovidiu Fira-Mladinescu, Diana Manolescu.

**Investigation:** Daniel Traila, Monica Steluta Marc.

**Resources:** Monica Steluta Marc, Camelia Pescaru.

**Supervision:** Ovidiu Fira-Mladinescu.

**Writing – original draft:** Daniel Traila.

**Writing – review & editing:** Ovidiu Fira-Mladinescu, Diana Manolescu.

## References

[1] Travis WD Costabel U Hansell DM . ATS/ERS Committee on Idiopathic Interstitial Pneumonias.An official American Thoracic Society/European Respiratory Society statement: update of the international multidisciplinary classification of the idiopathic interstitial pneumonias. Am J Respir Crit Care Med 2013;188:733–48. 24032382 10.1164/rccm.201308-1483ST PMC5803655

[2] Raghu G Remy-Jardin M Myers JL . American Thoracic Society, European Respiratory Society, Japanese Respiratory Society, and Latin American Thoracic Society.Diagnosis of idiopathic pulmonary fibrosis. An Official ATS/ERS/JRS/ALAT clinical practice guideline. Am J Respir Crit Care Med 2018;198:e44–68. 30168753 10.1164/rccm.201807-1255ST

[3] Raghu G Collard HR Egan JJ . An official ATS/ERS/JRS/ALAT statement: idiopathic pulmonary fibrosis: evidence-based guidelines for diagnosis and management. Am J Respir Crit Care Med 2011;183:788–824. 21471066 10.1164/rccm.2009-040GL PMC5450933

[4] Poletti V Ravaglia C Buccioli M . Idiopathic pulmonary fibrosis: diagnosis and prognostic evaluation. Respiration 2013;86:05–12. 10.1159/000353580 23816667

[5] Sauleda J Núñez B Sala E Soriano JB. Idiopathic pulmonary fibrosis: epidemiology, natural history, phenotypes. Med Sci (Basel) 2018;6:110. 10.3390/medsci6040110 PMC6313500 30501130

[6] Alba MA Flores-Suárez LF Henderson AG . Interstital lung disease in ANCA vasculitis. Autoimmun Rev 2017;16:722–9. 28479484 10.1016/j.autrev.2017.05.008 PMC6343660

[7] Mukhtyar C Guillevin L Cid MC . EULAR recommendations for the management of primary small and medium vessel vasculitis. Ann Rheum Dis 2009;68:310–7. 18413444 10.1136/ard.2008.088096

[8] Altschuler EL. Consideration of mycophenolate mofetil for idiopathic pulmonary fibrosis. Med Hypotheses 2001;57:701–2. 11918429 10.1054/mehy.2001.1437

[9] Liu GY Ventura IB Achtar-Zadeh N . Prevalence and clinical significance of antineutrophil cytoplasmic antibodies in North American patients with idiopathic pulmonary fibrosis. Chest 2019;156:715–23. 31181198 10.1016/j.chest.2019.05.014 PMC6859239

[10] Ando M Miyazaki E Ishii T . Incidence of myeloperoxidase anti-neutrophil cytoplasmic antibody positivity and microscopic polyangitis in the course of idiopathic pulmonary fibrosis. Respir Med 2013;107:608–15. 23434037 10.1016/j.rmed.2013.01.006

[11] Hozumi H Enomoto N Oyama Y . Clinical implication of proteinase-3-antineutrophil cytoplasmic antibody in patients with idiopathic interstitial pneumonias. Lung 2016;194:235–42. 26873743 10.1007/s00408-016-9851-x

[12] Kagiyama N Takayanagi N Kanauchi T . Antineutrophil cytoplasmic antibody-positive conversion and microscopic polyangiitis development in patients with idiopathic pulmonary fibrosis. BMJ Open Respir Res 2015;2:e000058. 10.1136/bmjresp-2014-000058 PMC4289718 25593704

[13] Hosoda C Baba T Hagiwara E . Clinical features of usual interstitial pneumonia with anti-neutrophil cytoplasmic antibody in comparison with idiopathic pulmonary fibrosis. Respirology 2016;21:920–6. 26994375 10.1111/resp.12763

[14] Katsumata Y Kawaguchi Y Yamanaka H. Interstitial lung disease with ANCA-associated vasculitis. Clin Med Insights Circ Respir Pulm Med 2015;9: (suppl 1): 51–6. 26448696 10.4137/CCRPM.S23314 PMC4583098

[15] Fernandez Casares M Gonzalez A Fielli M . Microscopic polyangiitis associated with pulmonary fibrosis. Clin Rheumatol 2015;34:1273–7. 24863847 10.1007/s10067-014-2676-1

[16] Watanabe T Minezawa T Hasegawa M . Prognosis of pulmonary fibrosis presenting with a usual interstitial pneumonia pattern on computed tomography in patients with myeloperoxidase anti-neutrophil cytoplasmic antibody-related nephritis: a retrospective single-center study. BMC Pulm Med 2019;19:194. 31675941 10.1186/s12890-019-0969-5 PMC6824021

